# A novel EIF3C-related CD8^+^ T-cell signature in predicting prognosis and immunotherapy response of nasopharyngeal carcinoma

**DOI:** 10.1007/s00432-023-05552-x

**Published:** 2024-02-24

**Authors:** Rui Li, Yikai Wang, Xin Wen, Binglin Cheng, Ruxue Lv, Ruzhen Chen, Wen Hu, Yinglei Wang, Jingwen Liu, Bingyi Lin, Haixiang Zhang, Enting Zhang, XinRan Tang

**Affiliations:** 1 Department of Radiation Oncology, Nanfang Hospital, Southern Medical University, 1838 North Guangzhou Avenue, Guangzhou, 510515 Guangdong Province China; 2 The First School of Clinical Medicine, Southern Medical University, Guangzhou, 510515 Guangdong Province China; 3 The First Affiliated Hospital, Sun Yat-Sen University, Guangzhou, 510080 Guangdong Province China

**Keywords:** Nasopharyngeal carcinoma, EIF3C, CD8^+^ T cells, Prognosis, Immunotherapy response

## Abstract

**Purpose:**

At present, dysfunctional CD8^+^ T-cells in the nasopharyngeal carcinoma (NPC) tumor immune microenvironment (TIME) have caused unsatisfactory immunotherapeutic effects, such as a low response rate of anti-PD-L1 therapy. Therefore, there is an urgent need to identify reliable markers capable of accurately predicting immunotherapy efficacy.

**Methods:**

Utilizing various algorithms for immune-infiltration evaluation, we explored the role of EIF3C in the TIME. We next found the influence of EIF3C expression on NPC based on functional analyses and RNA sequencing. By performing correlation and univariate Cox analyses of CD8^+^ Tcell markers from scRNA-seq data, we identified four signatures, which were then used in conjunction with the lasso algorithm to determine corresponding coefficients in the resulting EIF3C-related CD8^+^ T-cell signature (ETS). We subsequently evaluated the prognostic value of ETS using univariate and multivariate Cox regression analyses, Kaplan–Meier curves, and the area under the receiver operating characteristic curve (AUROC).

**Results:**

Our results demonstrate a significant relationship between low expression of EIF3C and high levels of CD8^+^ T-cell infiltration in the TIME, as well as a correlation between EIF3C expression and progression of NPC. Based on the expression levels of four EIF3C-related CD8^+^ T-cell marker genes, we constructed the ETS predictive model for NPC prognosis, which demonstrated success in validation. Notably, our model can also serve as an accurate indicator for detecting immunotherapy response.

**Conclusion:**

Our findings suggest that EIF3C plays a significant role in NPC progression and immune modulation, particularly in CD8^+^ T-cell infiltration. Furthermore, the ETS model holds promise as both a prognostic predictor for NPC patients and a tool for adjusting individualized immunotherapy strategies.

**Supplementary Information:**

The online version contains supplementary material available at 10.1007/s00432-023-05552-x.

## Introduction

Nasopharyngeal carcinoma (NPC), which originates from epithelium cells, is predominantly endemic to southeastern Asia, notably in south China’s Guangdong province, where the highest incidence has persisted without decline in the past 2 decades (Chen et al. 2019; Wei et al. 2010). With the infection with EPV prevalent in the NPC population, EBV-associated NPC is recognized as “immune-hot” tumors characterized by dense infiltration of immune cells in the tumor immune microenvironment (TIME), which is supposed to be beneficial for the efficacy of immunotherapy (Tsao et al. 2014; Wong et al. 2021). Therefore, the rise of an evolutionary wave of NPC immunotherapies, especially immune checkpoint inhibitors (ICIs) has become a research hotspot (Li et al. 2022a). Nevertheless, the response rates of immunotherapies in NPC remain unsatisfactory, with 33% of PD-L1-positive cases and 13% of PD-L1-negative cases (Xu et al. 2022; Ma et al. 2018). The immunoediting-based optimized neoantigen load (ioTNL) model has been reported to identify immunosuppressive tumor clones, and a subset of NPC subtypes (E-IS and A-IS) can predict the objective response rate and clinical outcomes of NPC patients’ immunotherapy to guide subsequent immunotherapeutic strategies (Su et al. 2022; Chen et al. 2021). Given the dynamic, complex, and interactive nature of the NPC TIME, which remains incompletely understood, it is imperative to identify additional biomarkers that can accurately predict the benefits derived from ICIs. Previous studies have proved that the infiltration levels of CD8^+^ T cells in the tumor microenvironment (TME) is correlated with ICI therapy response (Leun et al. 2020). With advancements in technology, including spatial transcriptomics and single-cell transcriptomics, novel insights are emerging, providing opportunities for more in-depth investigations into the heterogeneity and functional changes of CD8^+^ T cells, as well as the mechanisms of intercellular interactions within the TIME (Leun et al. 2020; Philip and Schietinger 2022). Researchers assessed the immune landscape of NPC through sequencing data and demonstrated that the dysfunctional and exhausted CD8^+^ T cells can create suppressive TME, thereby hindering long-term immunotherapy response in patients (Wong et al. 2021; Philip and Schietinger 2022). Recently, scRNA-seq data have been utilized to reveal features and dysfunctional statuses of CD8^+^ T cells in NPC patients. For example, research has shown that high expression of CD74 significantly increases the number of exhausted CD8^+^ T cells, while another study constructed an immune score of 36 genes to indicate the dysfunction of CD8^+^ T cells (Ka-Yue Chow et al. 2022; Jin et al. 2020). However, questions regarding the regulatory mechanisms of CD8^+^ T cells in the context of NPC immunotherapy response have yet to be answered. Therefore, identifying subtypes of CD8^+^ T cells along with their molecular mechanisms of NPC immunotherapy counts for much.

In the initiation stage of translation, a process for most translation regulators playing a role, eukaryotic translation initiation factors (EIFs) are essential. Furthermore, they have a significant impact on the expression of cancer genes (Sonenberg and Hinnebusch 2009). Among these factors, the core subunit C of eukaryotic translation initiation factor 3 (EIF3C) is involved in assembling the core EIF3 complex, which is essential for translation initiation. It has been demonstrated that the reduction of EIF3C expression inhibits the proliferation and metastasis of various cancer types through modulation of cell cycle progression (Sizova et al. 1998; Zhou et al. 2008; Emmanuel et al. 2013; Liu et al. 2020). So far, overexpressed EIF3C has been determined in a variety of tumors, such as ovarian cancer, colon cancer, renal cell carcinoma and cervical cancer, but few studies have mentioned NPC (Liu et al. 2020; Song et al. 2013; Fan et al. 2019; Hu et al. 2019). Zhao et al. have investigated the role of EIF3C in NPC by examining the expression levels of genes in apoptosis-related signaling pathways, and discovered that downregulation of EIF3C made an impact on the expression of phosphorylated P44/p42 MAPK, phosphorylated AKT, and phosphorylated SMad2, promoting apoptosis of pharyngeal squamous carcinoma cells by downregulating these genes’ expression (Zhao et al. 2022). However, there have been no research elucidating the regulatory role of EIF3C in the TIME and immunotherapy response prediction of NPC patients. In this study, in addition to investigating the role of EIF3C in NPC, we establish the relationship between EIF3C and infiltrated CD8^+^ T cells and construct the EIF3C-related CD8^+^ T-cell signature (ETS) in predicting the prognosis and immunotherapy response of NPC patients.

## Materials and methods

### Data collection and reprocessing

In this study, a training set consisting of RNA-seq data and corresponding clinical-pathologic data from 113 nasopharyngeal carcinoma (NPC) patient samples was acquired from the Gene Expression Omnibus (GEO) database (http://www.ncbi.nlm.nih.gov/gds/; GSE102349). The Illumina HiSeq 2000 sequencing platform was utilized in GSE102349. To validate our results, transcriptome data, mutation data, and corresponding clinical information from head and neck squamous cell carcinoma (HNSC) patients were obtained from The Cancer Genome Atlas (TCGA) database (https://portal.gdc.cancer.gov/exploration/), involving a total of 493 patient samples. In TCGA-HNSC, RNA-seq profiling was provided in fragments per kilobase million (FPKM). We transformed the FPKM values into the transcripts per kilobase million (TPM) values to reduce the biased values FPKM caused when comparing multiple samples (Vera Alvarez et al. 2019). Furthermore, single-cell RNA sequencing (scRNA-seq) data from 15 NPC patient samples and normal nasopharyngeal epithelial tissue from 1 patient were obtained from GEO dataset (GSE150430), utilizing the HiSeq X Ten sequencing platform.

### Analysis for the relationship between EIF3C and the TIME landscape

The “IOBR” R package was used to analyze immune infiltration (Zeng et al. 2021). Within this package, the “ESTIMATE” method was utilized to calculate the immune scores, stromal scores, and ESTIMATE scores (Yoshihara et al. 2013). In addition, we employed a number of algorithms including “MCPcounter” (Becht et al. 2016), “EPIC” (Racle et al. 2017), “quanTIseq” (Finotello et al. 2019), and “xCell” (Aran et al. 2017) to assess the abundance of immune cells and stromal cells in NPC microenvironment. Based on EIF3C’s expression and clinical data from GSE102349, we further utilized these immunocyte-infiltrating scores to respectively conduct correlation analysis and univariate Cox regression analysis. In the scoring and correlation analyses related to immune infiltration, we divided patients from TCGA-HNSC and 88 samples with survival information in GSE102349 into high (upper 25%) and low (lower 25%), as well as high (upper 25%) and low (lower 20%) groups based on the expression levels of EFI3C. And in univariate Cox regression analysis, we used the median of EIF3C expression to group 88 patients from GSE102349.

### Cell lines and cell culture

We selected two human NPC cell lines, HONE1 and SUNE1, which were both cultured in RPMI 1640 (Invitrogen, Grand Island, NY, USA) supplemented with 10% fetal bovine serum (FBS; Gibco, Grand Island, NY, USA). The human NPC cell line S18 was cultured in DMEM (Invitrogen) supplemented with 10% FBS. These cell lines were incubated in a humidified chamber with 5% CO_2_ at 37 °C.

### Transfection assay

We seeded and cultured these cells in six-well plates at 37 °C for 24 h. When the cells reached 50–70% confluence, they were infected with a small interfering EIF3C-1 RNA (si-eIF3c-1, siRNA: 5′-GCAGGACAACATTCAGCAT-3′), a small interfering eIF3c-2 RNA (si-eIF3c-2, siRNA: 5′-GCACACCTACTACAAGTTT-3′) and a negative control siRNA (si-SCR) which were all constructed by RiboBio (Guangzhou, China). Lipofectamine 3000 (Invitrogen) was used for transfections according to the manufacturer’s instructions. The efficiency of the transfections was investigated after 48–72 h using real-time PCR (RT-PCR).

### RNA extraction and RT-PCR

Total RNA from cultured cells was isolated with TRIzol reagent (Invitrogen). Then we performed reverse transcription of total RNA using reverse transcriptase (Promega, Madison, WI, USA) and random primers (Promega). SYBR Green–based (Invitrogen) RT-PCR analysis was carried out in a CFX96 Touch sequence detection system (Bio-Rad, Hercules, CA, USA). Real-time PCR primers for EIF3C and GAPDH were as follows: forward: 5′ TGAAGATTCGTGATGTCACCAAG-3′, reverse: 5′-AGATAGTCCTCTAGGTCAGCCA-3′; forward: 5′-GTCTCCTCTGACTTCAACAGCG-3′, reverse: 5′-ACCACCCTGTTGCTGTAGCCAA-3′. GAPDH was regarded as an endogenous control for all the genes. The 2^−ΔΔCT^ equation was utilized to calculate the relative gene expression.

### CCK8 and colony formation assays

HONE1 and SUNE1 cells were seeded at 1000 cells per well in 96-well plates after transfection. Cell viability was measured using a CCK8 assay (TargetMol, Shanghai, China) at five time points (0 h, 24 h, 48 h, 72 h, and 96 h, respectively) at OD450 nm. For the colony formation assay, HONE1 and SUNE1 cells were plated at 1000 cells per well in 6-well plates after transfection, and cultured for 14 days. Colonies were fixed with methanol/acetic acid (3:1, v/v), stained with 0.5% crystal violet, and counted under the inverted microscope.

### Transwell migration and invasion assays

For migration and invasion assays, we resuspended 5 × 10^4^ or 1 × 10^5^ transfected cells in serum-free medium and plated in the upper Transwell chamber (8-μm pores; Corning, Corning, NY, USA) with 8-mm pore size membrane with or without Matrigel (BD Biosciences, NJ, USA). Then the medium supplemented with 20% FBS was placed in the lower chamber. After 12 or 24 h of incubation, the cells that migrated or invaded through the upper membrane were fixed, stained with hematoxylin, and counted using an inverted microscope.

### RNA sequencing (RNA-seq)

RNA-seq was processed according to the instructions of NEBNext Ultra RNA Library Prep Kit for Illumina. Briefly, total RNA was isolated from EIF3C knock-down or control S18 cells using Trizol reagent. Poly(A) RNA was subsequently purified by PolyTtract mRNA Isolation System and used to generate cDNA libraries. All samples were sequenced on Illumina HiSeq X Ten platform and sequence reads were mapped to the human genome version hg38 by utilizing the Illumina sequence analysis pipeline. The average gene expression values of three independent studies were used for following analysis.

### Identification of CD8^+^ T-cell marker genes

The scRNA-seq data were analyzed via R package “Seurat” (Slovin et al. 2284). To ensure data quality, we filtered out cells with less than 201 or more than 9000 genes, as well as cells containing greater than 20% unique molecular identifiers (UMIs) originating from mitochondrial genome. Principal component analysis (PCA) and Uniform Manifold Approximation and projection (UMAP) were conducted to cluster cells in linear and nonlinear dimension reduction manners, respectively. During the process of screening marker genes, we applied log2 fold change > 1 and *p* value < 0.05 as filtering criteria.

### Construction and verification of the EIF3C-related CD8^+^ T-cell signature

In this study, we utilized the RNA sequencing (RNA-seq) profiling data from GSE102349 as the training set to examine the correlation between EIF3C and CD8^+^ T-cell marker genes using Spearman’s coefficient correlation. The prognostic values of the marker genes were evaluated by univariate Cox regression analysis using the “survival” and “survminer” R packages. Subsequently, we performed Lasso Cox regression analysis using the “glmnet” R package, with a parameter setting of alpha = 1. The formula of the risk score was constructed as follows: risk score = *β*1 × 1 + *β*2 × 2 + ··· + *βi* *xi*, where *xi* represents normalized expression of the candidate gene, and βi represents the corresponding coefficient derived from LASSO analysis. Kaplan–Meier method was employed for survival analysis, including overall survival (OS) and progression-free survival (PFS). Finally, the accuracy of our prognostic model was evaluated by calculating the area under the receiver operating characteristic curve (AUROC) using the “pROC” R package, and the enriched pathways in the low-risk group were analyzed by gene set enrichment analysis (GSEA) via “ClusterProfiler” and “enrichplot” R packages.

### Collection of data of immunological checkpoint inhibitor therapy

In this study, RNA-seq data and corresponding clinical–pathological information of patients who underwent anti-PD-1/PD-L1 treatment for melanoma were obtained from the GSE78220 (*n* = 26) and GSE91061 (*n* = 51) datasets. The platforms used for GSE78220 and GSE91061 were Illumina HiSeq 2000 and Illumina Genome Analyzer, respectively. Moreover, we obtained a urothelial carcinoma dataset, IMvigor, through the “IMvigor210CoreBiologies” R package, containing 348 samples with complete RNA-seq profiling and clinical characteristics (Balar et al. 2017). The count values in IMvigor were converted to FPKM, which were then transformed into TPM.

### Statistical analysis

Student’s *t* test or the Wilcoxon rank-sum test was employed for differential comparison between two groups. The significance of disparity was accessed through the log-rank test. In the GSE102349 and TCGA-HNSC cohorts used for survival analysis, patients were divided into high-risk and low-risk groups based on the median, while the GSE78220, GSE91061I, and Mvigor210 cohorts were stratified using their optimal cutpoints. All statistical analyses were conducted using R software (version 4.2.3) with statistically significance of *p* < 0.05.

## Result

### The correlation between EIF3C and infiltrated immunocytes

**Fig. 1:**
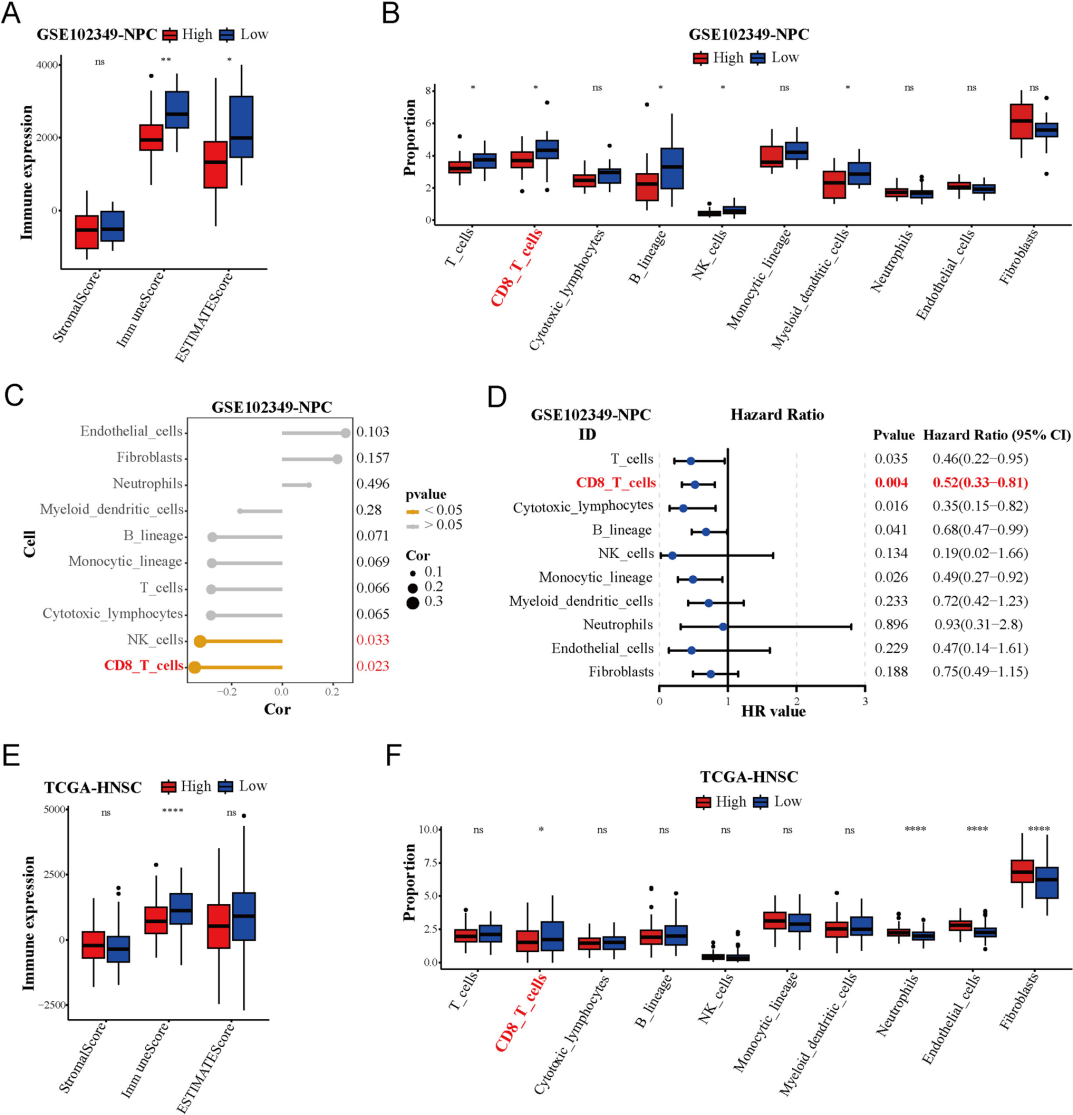
Function of the core subunit C of eukaryotic translation initiation factor 3 (EIF3C) in nasopharyngeal carcinoma (NPC) tumor microenvironment (TME). **A**, **E** Comparison of the immune-evaluation results in EIF3C-high and EIF3C-low groups by the ESTIMATE algorithm in GSE102348 and TCGA-HNSC cohorts (ns: not significant, *p < 0.05, **p < 0.01, ****p < 0.0001). **B**, **F** Comparison of the proportion of cells related to TME between EIF3C-high and EIF3C-low groups by the MCPcounter algorithm in GSE102349 and TCGA-HNSC cohorts (ns: not significant, *p < 0.05, ****p < 0.0001). **C** The spearman correlation analysis between immune cell infiltration levels (MCPcounter) and expression levels of EIF3C in GSE102349. **D** The univariate COX regression analysis of immune cell infiltration levels (MCPcounter) in GSE102349

To examine the impact of EIF3C in the TIME, we first applied MCPcounter and ESTIMATE algorithms in GSE102349 cohort and found that low expression of EIF3C was associated with higher immune scores and T cells, CD8 + T cells, B lineage, NK cells, myeloid dentritic cells were more abundant in the EIF3C-low group (Fig. 1A, B), suggesting that NPC patients with lower EIF3C expression may possess a more favorable TIME. To investigate the most relevant immune cells for EIF3C, correlation analysis and univariate COX regression analysis were conducted on GSE102349-MCPcounter. The result showed that the levels between CD8 + T cells infiltration and EIF3C were most correlated, and the level of CD8 + T cells infiltration was the most important protective factor for PFS in NPC (Fig. 1C, D). To further validate our findings, we performed comprehensive analysis of TIME to analyze the association with EIF3C and CD8 + T cells infiltration and the CD8 + T cells showed the same performance like MCPcounter in GSE102349 (Fig. S1A–E, F). More importantly, CD8 + T cells were also enriched in EIF3C-low patients in TCGA-HNSC cohort and was similarly negatively correlated with EIF3C in the TCGA-HNSC and GSE53819 cohorts (Fig. 1E, F; Fig. S1G, H). As CD8^+^ T cells are known for their exceptional cytotoxic activity to tumor cells (St Paul and Ohashi 2020), overexpression of EIF3C in NPC patients might hinder anti-tumor immune response by potentially suppressing CD8^+^ T cells. Taken together, our findings suggest that EIF3C affects the infiltration of CD8^+^ T cells, which is crucial for its regulatory effect on the TIME of NPC patients.

### EIF3C facilitated NPC cell proliferation, migration, and invasion

**Fig. 2:**
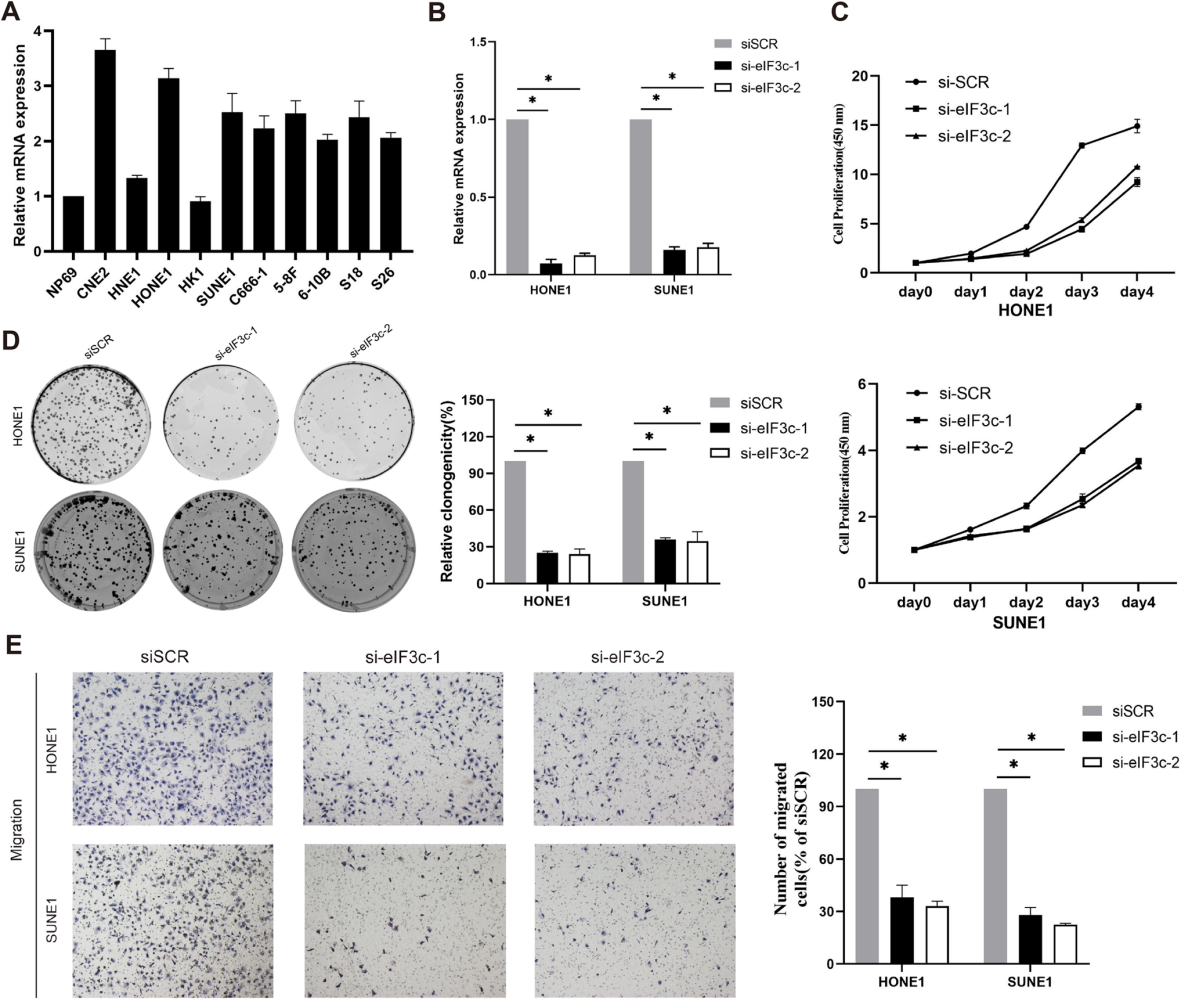
Functional analyses for the role of EIF3C in cell proliferation, migration, and invasion. **A** Relative EIF3C expression in NPC cell lines. **B** The transfection efficiency of si-eIF3c was verified in HONE1 and SUNE1 cells (*p < 0.05). **C**, **D** CCK8 and colony formation assays showed that EIF3C facilitated cell proliferation in HONE1 and SUNE1 (*p < 0.05). **E** Representative and quantified results of Transwell migration and invasion assays in HONE1 and SUNE1 cells with transfection of si-eIF3c or si-SCR (*p < 0.05)

To explore the biological role of EIF3C, we conducted functional analyses including cell proliferation, migration, and invasion. We observed that the endogenous EIF3C expression was sharply increased in NPC cell lines compared with a nasopharyngeal epithelial cell line (Fig. 2A). Then HONE1 and SUNE1 cell lines were selected for further experiments. We transfected si-eIF3c into HONE1 and SUNE1 cells to reduce EIF3C expression. Simultaneously, siSCR was transfected into these cells as a control group. Then we employed quantitative real-time PCR to validate the efficiency of transfection and confirmed that the EIF3C inhibitor significantly suppressed expression of EIF3C in HONE1 and SUNE1 cells (Fig. 2B).

According to CCK8 and colony formation assays, we found that the knock-down of EIF3C suppressed HONE1 and SUNE1 cell proliferation (Fig. 2C, D). Furthermore, we performed Transwell migration and invasion experiments, and the results showed that the migratory and invasive capacities of HONE1 and SUNE1 cells were also remarkably inhibited by the downexpression of EIF3C (Fig. 2E). To, sum up, these data indicated EIF3C as a tumor-promoting factor.

### Transcriptional response in NPC cells to the transfection of si-eIF3c

**Fig. 3:**
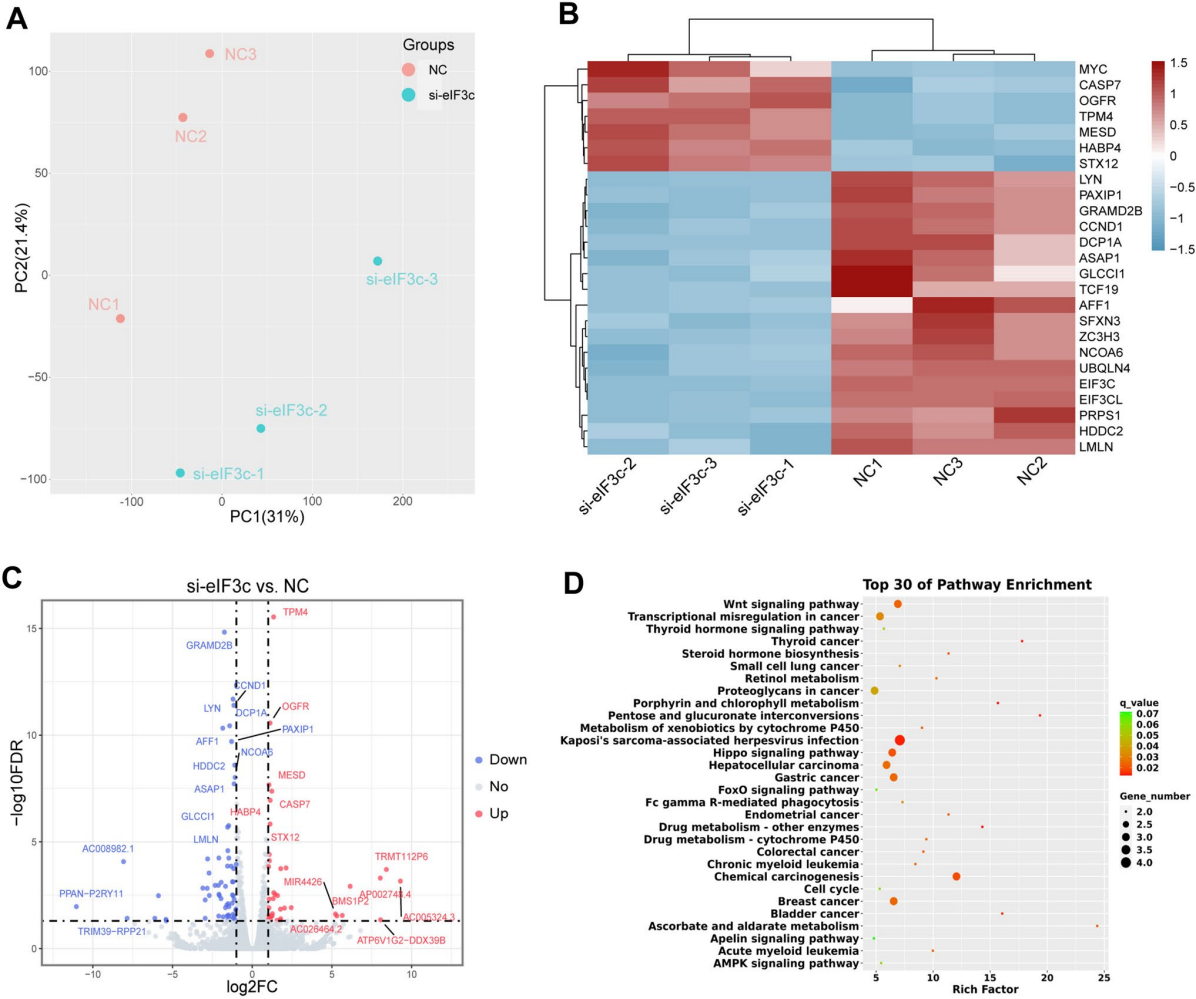
Transfection of si-eIF3c induces a transcriptional response in S18 cells. **A** PCA analysis of all six samples, grouped by si-SCR (NC, pink) and si-eIF3c (blue). **B** Heatmap of top 25 DEGs ranked by p value and clustered by treatment. **C** Volcano plot of DEGs, which were colored by upregulated (red) and downregulated (blue) DEGs. Genes with │log2FC│ > 1 and FDR < 0.05 were regarded as DEGs. **D** A bubble plot of top 30 enriched KEGG pathways in the order of q-value using 106 DEGs

To reveal the functional mechanism of EIF3C, we next applied bulk RNA-sequencing to S18 cells with (*n* = 3) or without (*n* = 3) transfection of si-eIF3c. Based on data of gene expression, we carried out principal component analysis (PCA) and found samples clustered by treatment (Fig. 3A). Then we screened 106 differentially expressed genes (DEGs), top 25 of which are presented in Fig. 3B. Since false discovery rates (FDRs) of EIF3C (log_10_FDR = − 53.433, log_2_FC = − 2.332), EIF3CL (log_10_FDR = − 52.304, log_2_FC = − 2.321), and MYC (log_10_FDR = − 24.189, log_2_FC = 2.603) far exceeded FDRs of other DEGs, there were only 41 out of 42 upregulated and 62 out of 64 downregulated genes exhibited in Fig. 3C. Since the top 25 of DEGs were almost genes related to the occurrence and development of various tumors, to explore the function of these DEGs, we performed KEGG enrichment analysis and further selected top 30 enriched pathways identified to be associated with DEGs using all these DEGs at 5% FDR. As shown in Fig. 3D, besides many pathways related to a variety of human cancers, two NPC related pathways, the Wnt signaling (Rich Factor = 7.165, *p* = 0.008, FDR = 0.052) and Hippo signaling (Rich Factor = 7.804, *p* = 0.006, FDR = 0.049), were relevant to DEGs (Wu et al. 2022a; Peng et al. 2022). What iss more, the activation of Wnt signaling pathway and deregulation of Hippo signaling pathway have been proved to hinder the development and functioning of effector CD8^+^ T cells in many tumors (Takeuchi et al. 2021; Gattinoni et al. 2009; Mohajan et al. 2021; Du et al. 2018), which might be two crucial ways for EIF3C to exert its immunosuppression effect. In brief, these results derived from sequencing revealed that the variation in EIF3C expression levels had an impact on the progression of NPC.

### Construction of EIF3C-related CD8^+^ T-cell signatures

**Fig. 4:**
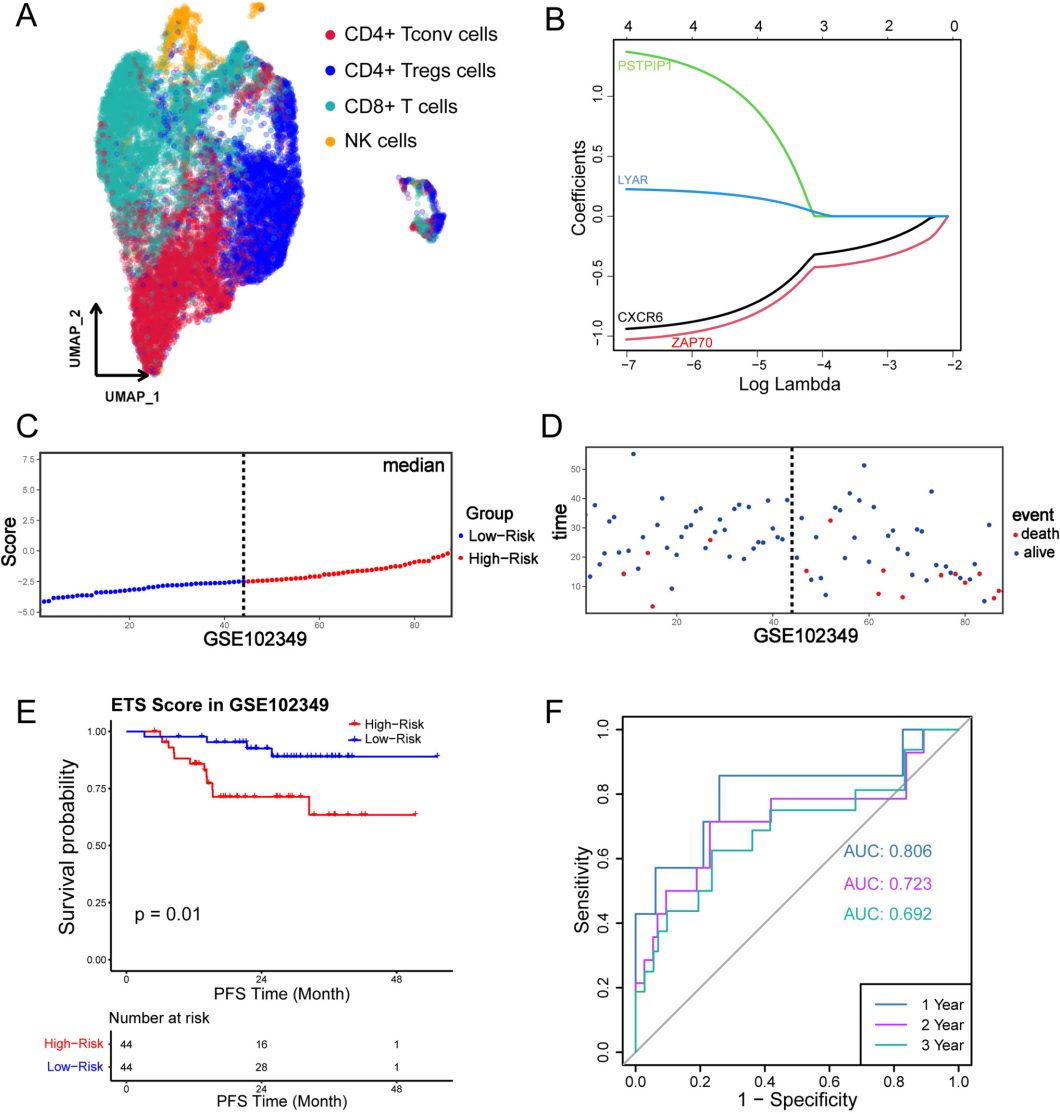
Construction of the EIF3C-related CD8^+^ T-cell signature (ETS) in GSE102349. **A** UMAP plot showed cell types in the GSE150430 dataset. **B** Coefficients of candidate genes were selected regarding lambda by lasso regression. Each curve meant a predictor. **C** NPC patients in GSE102349 were separated into two groups according to the median of the risk score. **D** The distribution of patients’ survival time and risk score in GSE102349. **E**, **F** Kaplan–Meier curves of PFS between the high- and low-risk groups, and the 1-, 2-, and 3-year area under the receiver operating characteristic (AUROC) curves depicted the performance of ETS for prognostic prediction efficacy in GSE102349

To further explore the clinical value and molecular mechanism underlying the changes in CD8^+^ T-cell activity influenced by EIF3C, we identified 210 CD8^+^ T-cell related marker genes (Fig. 4A, Fig. S2A, Table S1) from a NPC scRNA-seq dataset (GSE150430) using FindAllMarkers analysis. Among them, 21 genes exhibited a significant correlation with EIF3C (*p* < 0.05) in GSE102349 dataset (Table S2). Then we still utilized GSE102349 cohort as a training set to estimate the prognostic values of the 21 EIF3C-related genes, and eventually got 4 genes (*CXCR6, ZAP70, PSTPIP1,* and *LYAR*) contributing to the OS by the univariate COX regression analysis (Fig. S2B). Given that the four candidate genes could be used to construct a prognostic model, we next carried on the lasso regression analysis to calculate the corresponding coefficient for each gene (Fig. 4B, Fig. S2C). And the prognostic EIF3C-related CD8^+^ T cell signature (i.e., ETS) was created whose risk score should follow this formula: ETS risk score = *CXCR6* expression × (− 0.9391) + *ZAP70* expression × (− 1.0279) + *PSTPIP1* expression × (1.3725) + *LYAR* expression × (0.2262) (Table 1).

**Table 1:** Genes and corresponding coefficients in the GSE102349 cohort for ETS

Gene	Coefficient
CXCR6	− 0.939117253
ZAP70	− 1.027889222
PSTPIP1	1.372532002
LYAR	0.226168806

Patients were divided into high- (*n* = 44) and low-risk (*n* = 44) groups by the median of risk scores (Fig. 4C). Analysis of the distribution of ETS risk scores revealed superior survival outcomes for the low-risk group compared to the high-risk group (Fig. 4D). We observed the significantly longer progression-free survival observed in patients with low risk through survival analysis (*p* = 0.01, Fig. 4E). Moreover, the AUROCs of 1-year, 2-year, and 3-year survival were 0.806, 0.723, and 0.692, respectively (Fig. 4F), indicating a favorable performance of the ETS risk score in predicting the prognosis of NPC patients.

### Validation of ETS risk score in TCGA cohort

**Fig. 5:**
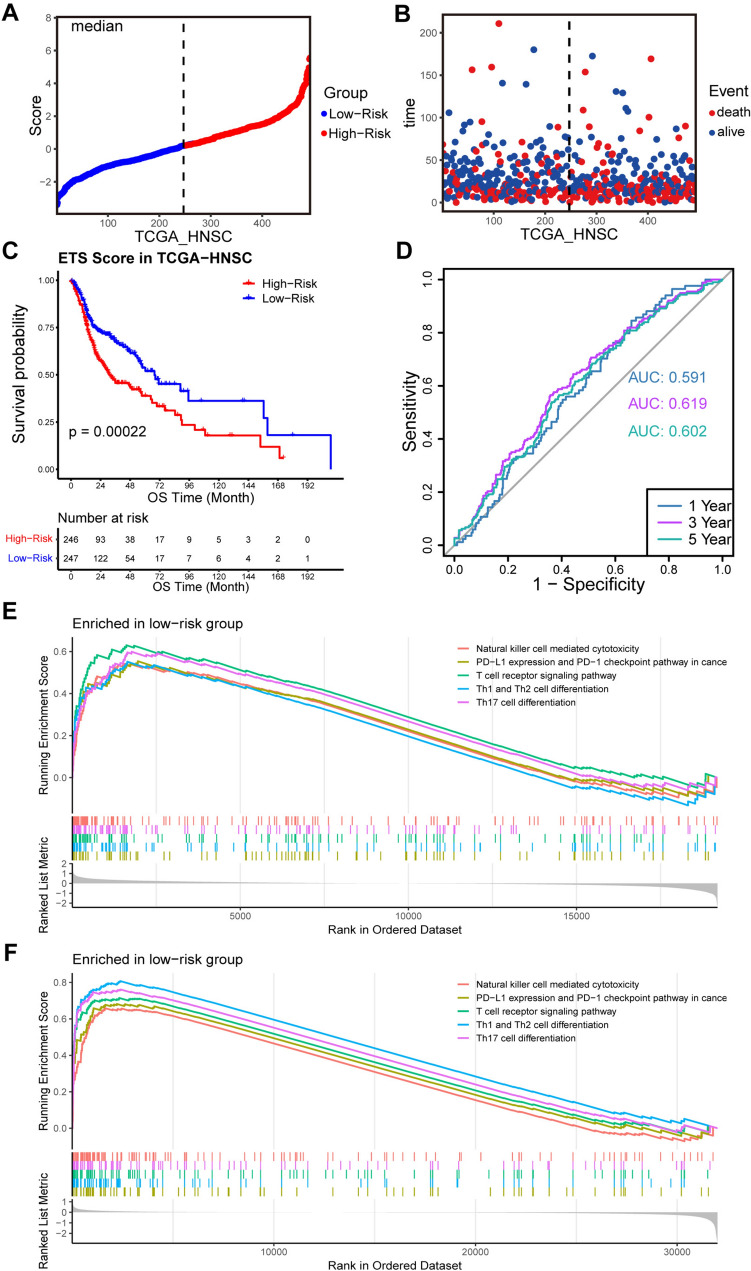
Validation of the prognostic values of ETS risk score in the validation set. **A** NPC patients in TCGA-HNSC were divided into two groups according to the median of the risk score. **B** The distribution of patients’ survival time and risk score in TCGA-HNSC. **C**, **D** Kaplan–Meier curves of OS between the high- and low-risk groups, and the AUROC for predicting 1-, 3-, and 5-year OS showed the performance of ETS for prognostic prediction efficacy in TCGA-HNSC. **E**, **F** GSEA plot showed five immune-related pathways were enriched in both GSE102349 and TCGA-HNSC cohorts

To examine the robustness of ETS as a prognostic predictor, we utilized the TCGA-HNSC cohort as a validation set. The procedure for survival analysis was analogous to that of the training set. First, the ETS risk scores were calculated for each patient, and the patients were then classified into high- (*n* = 246) and low-risk (*n* = 247) groups based on the median ETS score (Fig. 5A). As observed in the training set, the low-risk patients in the validation set also demonstrated better overall survival than their high-risk counterparts (Fig. 5B). Consistent with this observation, a significant difference in overall survival was found between the two groups, with patients in the low-risk group exhibiting longer survival time (*p* < 0.001, Fig. 5C). Moreover, the ETS risk score exhibited favorable reproducibility in the validation set, with AUROCs of 0.591, 0.619, and 0.602 for predicting 1-year, 3-year, and 5-year survival, respectively (Fig. 5D). These findings provide further confirmation of the effectiveness of ETS as a prognostic predictor.

In addition, GSEA for GSE102349 and TCGA-HNSC datasets were conducted for the further research, which showed that there were five immune-related pathways (PD − L1 expression and PD − 1 checkpoint pathway in cancer, T cell receptor signaling pathway, Th1 and Th2 cell differentiation, Natural killer cell mediated cytotoxicity, and Th17 cell differentiation) enriched in both two low-risk groups (Fig. 5E, F). These findings provide additional support for the validity of the ETS risk score as a prognostic predictor and offer guidance for future research aimed at elucidating the mechanisms underlying the association between EIF3C and immune function in NPC patients.

### Identifying the ETS risk score as an independent predictor

**Fig. 6:**
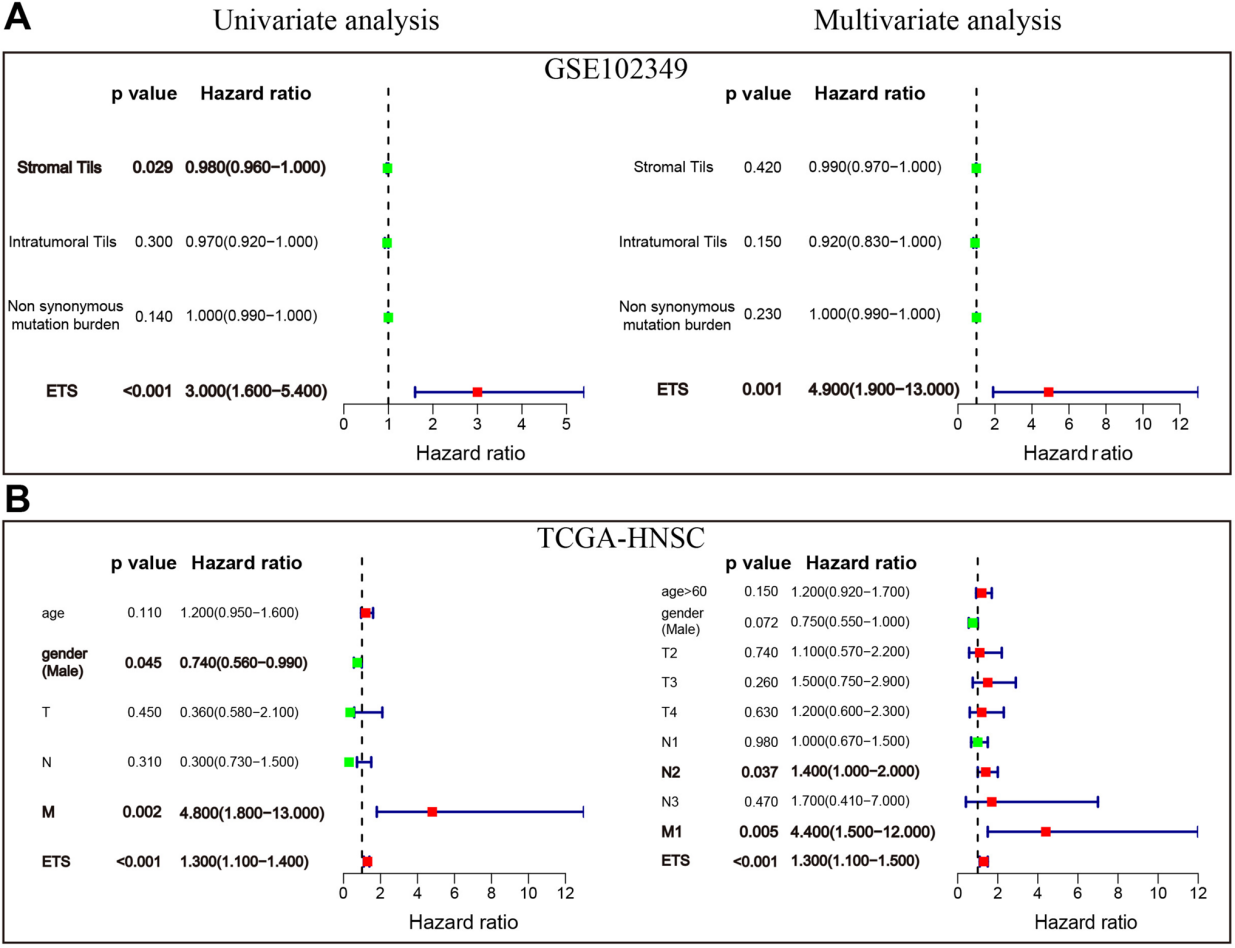
Identification of ETS risk score as an independent prognostic factor. Univariate and multivariate Cox regression analysis of the ETS risk score in the training (**A**) and validation (**B**) datasets

To evaluate the prognostic value of the ETS risk score in NPC patients as compared to other clinical characteristics, both univariate and multivariate Cox regression analyses were conducted. The univariate analysis revealed that the low-risk ETS score was significantly associated with good survival outcomes. Specifically, the hazard ratios were 3.000 (95% CI = 1.600–5.400, *p* < 0.001; Fig. 6A) in the GSE102349 training set and 1.300 (95% CI = 1.100–1.400, *p* < 0.001; Fig. 6B) in the TCGA validation set. And the multivariate analysis showed that even after adjusting for other clinical features, the association between the low-risk ETS score and favorable survival outcomes remained significant with a hazard ratio of 4.900 (95% CI = 1.900–13.000, *p* = 0.001; Fig. 6A) in GSE102349 and a hazard ratio of 1.300 (95% CI = 1.100–1.500, *p* < 0.001; Fig. 6B) in TCGA-HNSC. Based on the above evidence, it can be concluded that the ETS risk score is most likely an independent prognostic factor for NPC patients.

### The prognostic value of ETS for immunotherapy

**Fig. 7:**
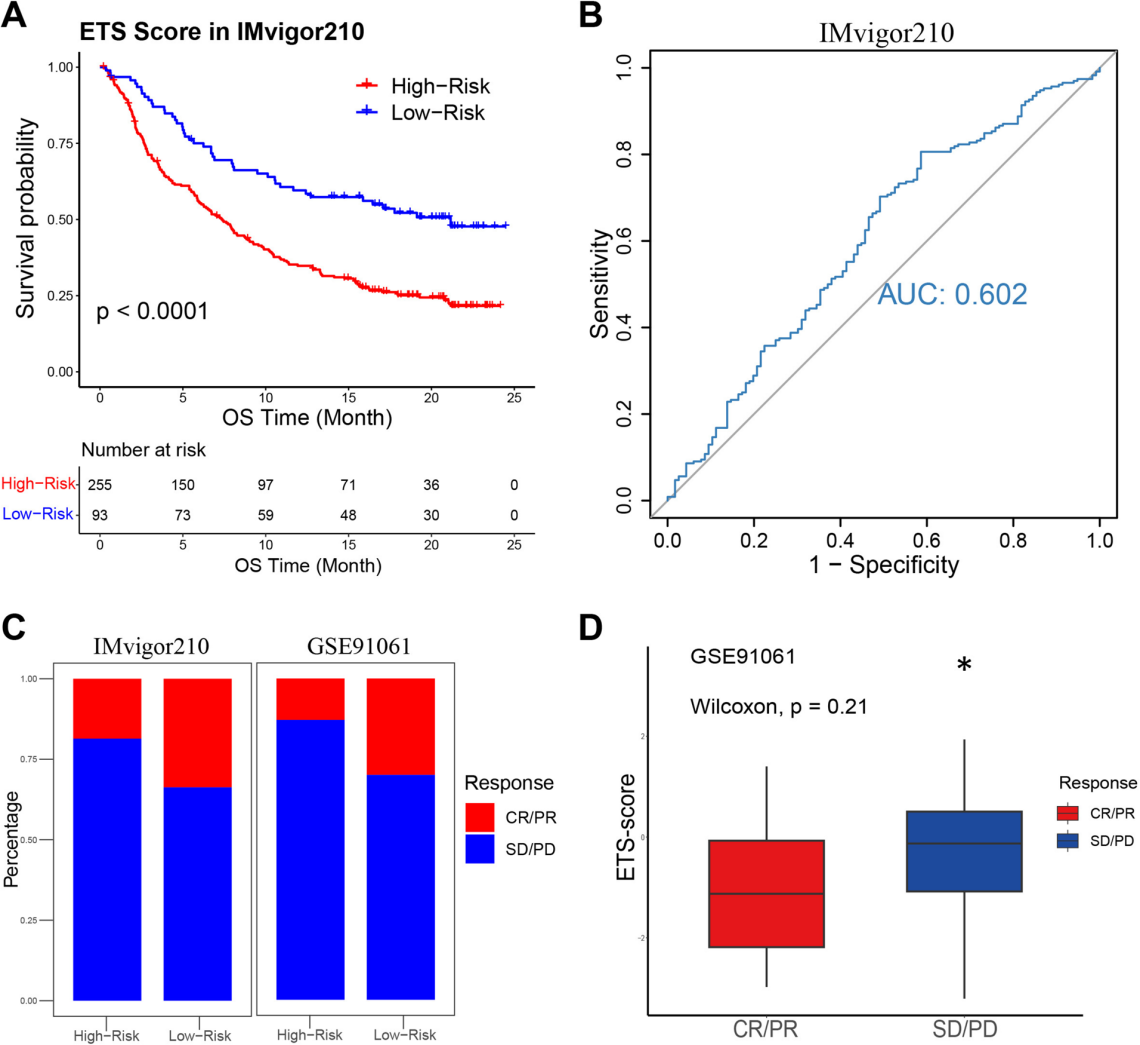
Performance of ETS for patients with immunotherapy. **A**, **B** Kaplan–Meier curves of OS between the high- and low-risk groups, and the AUROC depicted the performance of ETS for predicting immunotherapy efficacy in the IMvigor210 cohort. **C**, **D** Responses of high- and low-risk patients from IMvigor210 and GSE91061 cohorts to anti-PD-L1 therapy [complete response (CR), progressive disease (PD), partial response (PR), and stable disease (SD); *p < 0.05]

According to the correlation between ETS and immune status, we next examine the prognostic value of the ETS risk score for immunotherapy. Immune checkpoint inhibitors, particularly anti-PD-1/PD-L1 immunotherapy, are increasingly used as a promising cancer treatment strategy (Wu et al. 2022a). We assessed the effectiveness of our ETS risk score in prognostic prediction for patients receiving anti-PD-1/PD-L1 therapy using the data from GSE78220, GSE91061, and IMvigor210 cohorts. Similarly to our previous findings, low-risk patients who had received immunotherapy also had longer OS than high-risk patients after the same treatment in all cohorts (Fig. 7A, Fig. S3A, C). And AUROC values were 0.726, 0.585, and 0.602 for GSE78220, GSE91061, and IMvigor210 cohorts, respectively (Fig. 7B, Fig. S3B, D). Moreover, a favorable immunotherapeutic response was observed in low-risk patients in the GSE91061 and IMvigor210 cohorts (Fig. 7C, D), thus confirming the relationship between low-risk patients and better survival outcomes following treatment. Therefore, we came to a conclusion that the ETS risk score also owned important value to predict prognosis of the patients after immunotherapy.

## Discussion

In this study, we first identified the role of EIF3C in the TIME of NPC through many algorithms, involving ESTIMATE, EPIC, quanTIseq, xCell, and MCPcounter, to evaluate the abundance of immune cells and stromal cells in NPC TIME. Our analysis revealed that overexpression of EIF3C has an immunosuppressive effect on infiltrating immune cells, particularly CD8^+^ T cells, an immune cell type which was also identified as an important prognostic factor for NPC patients. Moreover, functional assays and sequencing results stated EIF3C’ facilitation on NPC progression, also reinforcing our viewpoint on the role of EIF3C in NPC TIME.

By analyzing scRNA-seq data, we confirmed 21 EIF3C-related CD8^+^ T-cell marker genes and then selected 4 of them (*CXCR6, ZAP70, PSTPIP1,* and *LYAR*) to build the ETS prognostic model. It has been proven that CXCR6 plays a prominent role in CD8 + T-cell enrichment and maintenance in various tissues including NPC, which was previously attributed to its nuclear factor NF-kB activation and CXCR6-mediated cellular interactions such as IL-15 trans-presentation via IL-15Rα (Parsonage et al. 2012; Pilato et al. 2021). Similarly, as a predominant component of the TCR signaling pathway, ZAP70 is essential for CD8 + T-cell proliferation and maturation by activating NF-kB (Wang et al. 2010; Hu et al. 2018). And in some reports, silencing ZAP70 has been demonstrated to significantly suppress the anti-tumor capability of CD8 + T cells (Hu et al. 2018; Cheng et al. 2023). And PSTPIP1, upregulated in a number of tumors, has been determined to undergo a dephosphorylation process to form the CD2-PSTPIP complex, which inactivates most src kinases and then inhibits the activation of CD8 + T cells (Li et al. 2022b; Bai et al. 2001). As for LYAR, a nucleolar oncoprotein originally isolated from T-cell leukemia line, it has been reported to contribute to worse survive as well as less CD8 + T-cell infiltration in cancer, and own a negative correlation with the degree of CD8 + T-cell exhaustion in TIME, so we deduce that LYAR may act as an obstacle to the maturation of CD8 + T cells (Su et al. 1993; Baitsch et al. 2011; Sun et al. 2021; Wang et al. 2021). The coefficients of our model were in line with previous findings, corroborating the rationality of our ETS risk score. In subsequent validation analysis, this model also represented good performance in predicting prognosis of NPC patients. Moreover, ETS proved to be an effective predictor of immunotherapy response across three verification datasets.

CD8^+^ T cells are key effector immune cells to eliminate malignant cells in the TME. Nevertheless, after long-term antigen exposure, T cells turn toward differentiation, and then eventually change into a dysfunctional and exhausted phenotype (Verdon et al. 2020; McLane et al. 2019). Currently, there is limited research exploring the effect of EIF3 subunits on the TIME, and there is no EIF3C-related research on T cells specifically because of the complex structure of the EIF3 complex. Therefore, analyzing the process of EIF3C affecting CD8^+^ T cells by combining multiple molecules or cells is necessary. It has been discovered that EIF3D plays its part of cap binding in promoting the development of regulatory T (Treg) cells by binding to DAP5, while the ligand–receptor interactions between Treg cells and exhausted T cells (Tex), such as CCL4-CCR8 and TTGAL-ICAM1, facilitate the formation of an immune-suppressive microenvironment and the depletion of CD8^+^ T cells (Liu et al. 2021; Volta et al. 2021). Based on the role of EIF3D mentioned above and the capability of EIF3C in recruiting EIF3D for the EIF3 complex, we may deduce a pathway about EIF3C affecting CD8^+^ T cells (Zhou et al. 2008), which also accords with the distribution of the certain proportion of Treg cells in our scRNA-seq data analysis (Fig. 4A). Moreover, an earlier study verified that EIF3B, as a key gene of the subtype of melanoma with the worst prognosis, has the ability of inducing immune-depletion and reducing CD8^+^ T-cell infiltration (Wu et al. 2022b). Although EIF3B has been confirmed as a scaffold connecting EIF3C and other EIF3 subunits, further exploration is needed to determine whether EIF3C can exert its influence on CD8^+^ T cells through EIF3B (Zhou et al. 2008). In our study, the ETS risk score, a model derived from EIF3C and CD8^+^ T cells, represented a significant reduction in PFS and OS for high-risk patients, possibly due to the lower immune scores and decreased CD8^+^ T-cell infiltration associated with high EIF3C expression.

In our study, we conducted GSEA on both training and validation cohorts and identified five immune-related pathways enriched in low-risk patients. Among these pathways, except for three pathways associated with CD4^+^ T cells, one of the remaining two is the T cell receptor (TCR) signaling pathway which is necessary for activating T cells by combining EIF3 and mRNAs of TCR subunits (Silva et al. 2021), another one is about the increased expression of PD-L1. To evaluate the efficacy of our model in predicting the prognosis of patients undergoing anti-PD-1/PD-L1 therapy, we utilized three datasets and concluded that low-risk patients exhibited longer overall survival rates and a more favorable response to immunotherapy. Similar to EIF3C, the expression of EIF3B is negatively related to the infiltration of immunocytes and represents a poorer prognosis (Wu et al. 2022b). Meanwhile, the patients with lower EIF3B expression have been proven to get better response to anti-PD-1 treatment, which is approximately the same as the response proportion of EIF3C in this study (Wu et al. 2022b). These findings lend support to the predictive capacity of our model and suggest that further investigation into the interaction between EIF3C and EIF3B is essential.

There were several limitations in our research. First, while our model performed well on training and validation cohorts, further examinations of additional datasets are necessary to enhance persuasiveness and generalizability of our findings. Second, as a retrospective study, our research needs experimental verification to explore more exact molecular mechanism of EIF3C functioning on CD8^+^ T cells and the TIME, which is also an important aspect of our future research.

## Conclusion

In summary, our study has identified EIF3C as a key regulator of CD8^+^ T cell distribution within the TIME of NPC. The ETS risk score constructed in our model exhibited strong predictive utility for patient prognosis and immunotherapy response. These findings offer valuable insights into potential prognostic strategies for NPC and illuminate a novel mechanism regarding the regulatory effects of EIF3C on the tumor immune microenvironment.

### Supplementary Information

Below is the link to the electronic supplementary material.

Supplementary file1 (PDF 1701 KB)

Supplementary file2 (CSV 11 KB)

Supplementary file3 (CSV 1 KB)

## Data Availability

Publicly available data were analyzed in this study. These data can be found here: TCGA; GSE102349; GSE150430; GSE78220; GSE91061; R package “IMvigor210CoreBiologies”.
