# Supplementary material for: A novel EIF3C-related CD8+ T-cell signature in predicting prognosis and immunotherapy response of nasopharyngeal carcinoma
Source: J Cancer Res Clin Oncol. 2024 Feb 24;150(2):103. doi: 10.1007/s00432-023-05552-x (PMC10894114; doi:10.1007/s00432-023-05552-x)
Supplement: Supplementary file 1 — Supplementary file1 (PDF 1701 KB) [file 432_2023_5552_MOESM1_ESM.pdf]

A novel EIF3C-related CD8^{+} T-cell signature in predicting prognosis and immunotherapy response of nasopharyngeal carcinoma

Journal of Cancer Research and Clinical Oncology
Supplementary Information

Rui Li1*, Yikai Wang1*, Xin Wen1,3*, Binglin Cheng1, Ruxue Lv1, Ruzhen Chen1, Wen Hu1, Yinglei Wang2, Jingwen Liu2, Bingyi Lin2, Haixiang Zhang2, Enting Zhang2, XinRan Tang1

1 Department of Radiation Oncology, Nanfang Hospital, Southern Medical University, Guangzhou 510515, Guangdong Province, China

2 The First School of Clinical Medicine, Southern Medical University, Guangzhou 510515, Guangdong Province, China

3 The First Affiliated Hospital, Sun Yat-sen University, Guangzhou, 510080, Guangdong Province, China

*These authors contributed equally to this study.

Correspondence: XinRan Tang

E-mail: tangxran@163.com

Institution: Department of Radiation Oncology, Nanfang Hospital, Southern Medical University, 1838 North Guangzhou Avenue, Guangzhou 510515, China

Fig S1 Comprehensive analysis of TIME to analyze the association with EIF3C and CD8+ T cells infiltration

Fig S2 Construction of the EIF3C-related CD8+ T-cell signature

Fig S3 Validation of the prognostic values of risk score in patients with anti-PD-L1 therapy

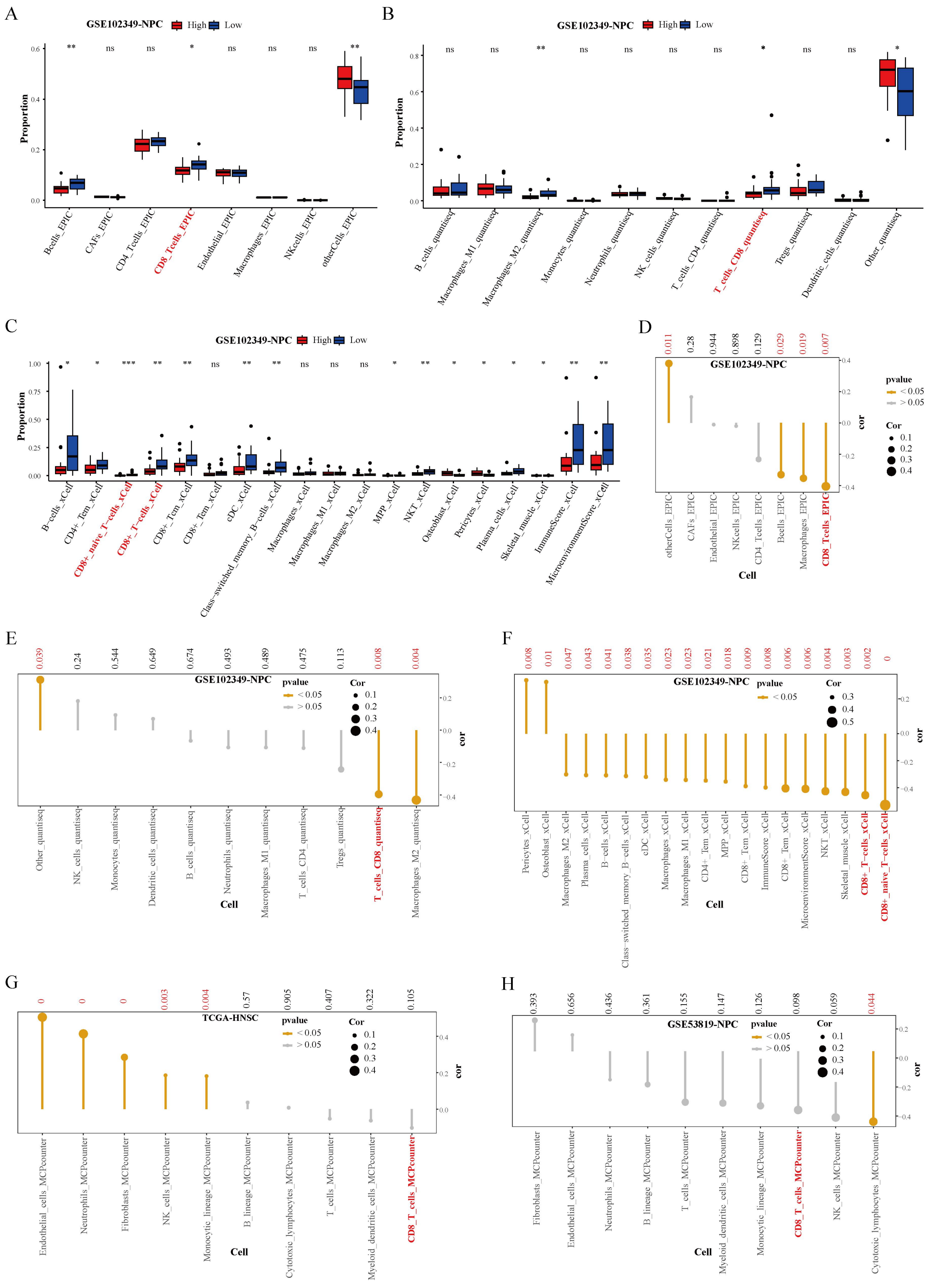

Fig S1 Comprehensive analysis of TIME to analyze the association with EIF3C and CD8+ T cells infiltration. (A, B, C) Comparison of the proportion of cells related to TME between EIF3C-high and EIF3C-low groups by the EPIC algorithm (A), quantiseq algorithm (B), Xcell algorithm (C) in GSE102349 (ns: not significant, *p < 0.05, **p < 0.01, ***p < 0.001). (D, E, F) The spearman correlation analysis between immune cell infiltration levels of the EPIC

algorithm (D), quantiseq algorithm(E), Xcell algorithm (F) and expression levels of EIF3C in GSE102349. (G,H) The spearman correlation analysis between immune cell infiltration levels (MCPcounter) and expression levels of EIF3C in TCGA-HNSC(G) and GSE53819(H)

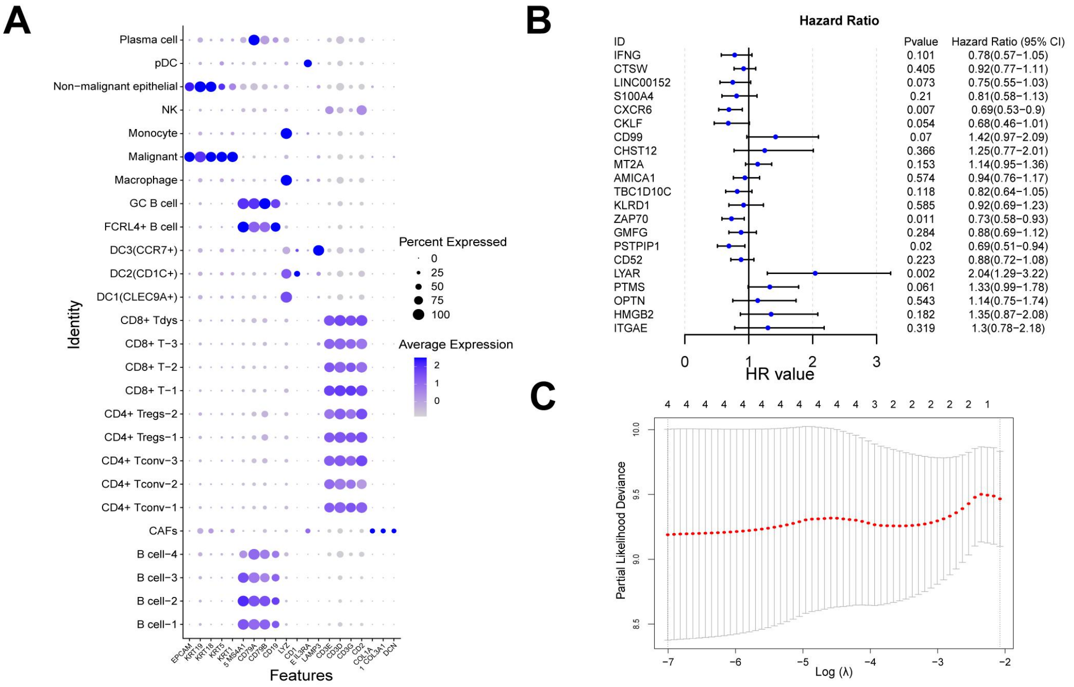

Fig S2 Construction of the EIF3C-related CD8+ T-cell signature. (A) Identification marker genes of different cell types in the GSE150430 dataset. (B) Candidate genes for ETS was selected by univariate Cox regression analysis in the training set. (C) Lambda distribution of lasso regression. The left line indicated the optimal value (log lambda = -7)

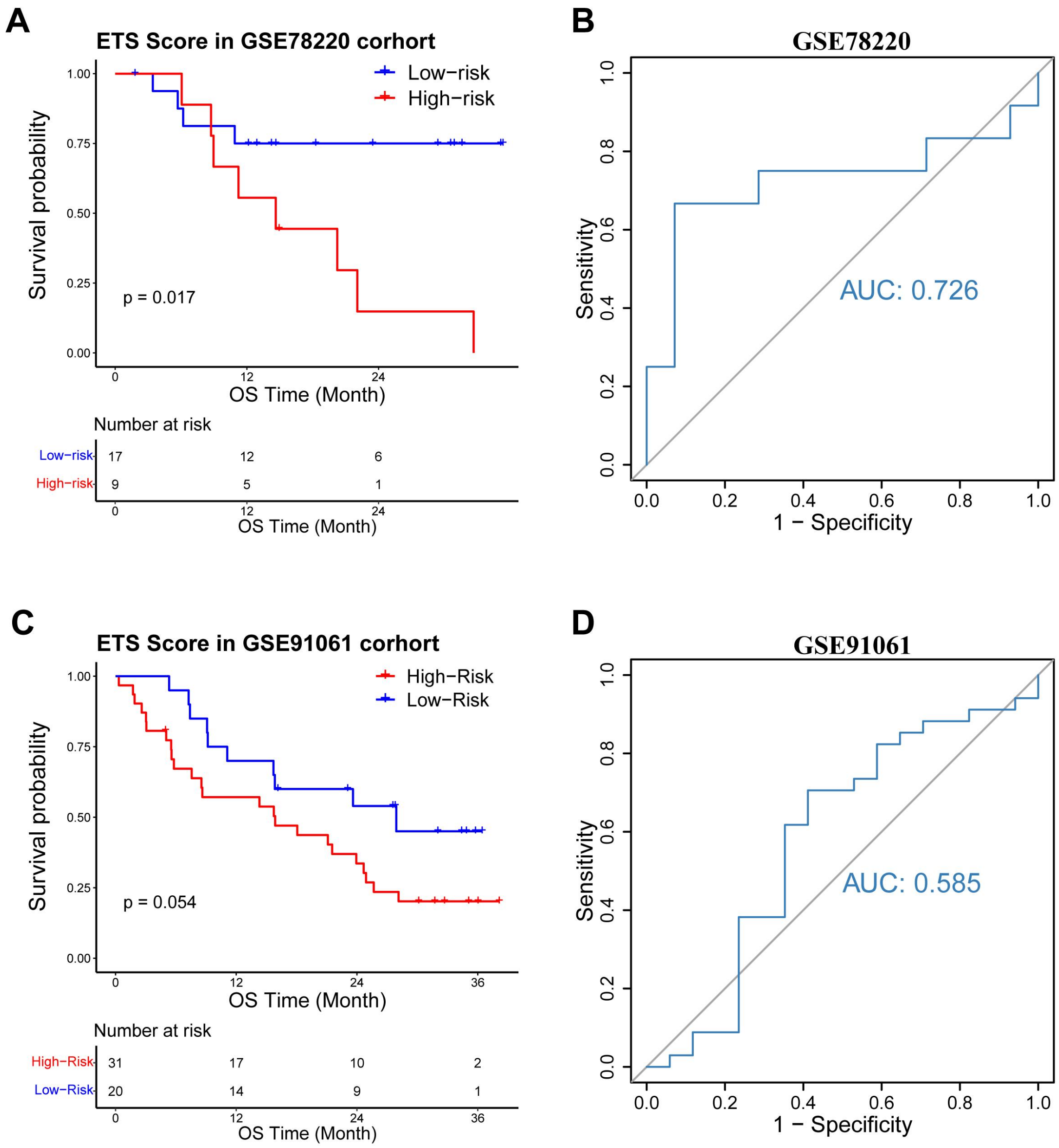

Fig S3 Validation of the prognostic values of risk score in patients with anti-PD-L1 therapy. Kaplan-Meier curves of OS between the high- and low-risk groups, and the area under the receiver-operating characteristic (AUROC) curves depicted the performance of ETS for predicting immunotherapy efficacy in GSE78220 (A-B) and GSE91061 (C-D), respectively
